# Bis(2-chloro­benz­yl)dimethyl­ammonium bromide

**DOI:** 10.1107/S160053680902159X

**Published:** 2009-06-13

**Authors:** Tariq Mahmud, Javed Iqbal, Mark R. J. Elsegood, Vickie McKee

**Affiliations:** a Institute of Chemistry, University of the Punjab, Lahore 54590, Pakistan; b Chemistry Department, Loughborough University, Loughborough LE11 3TU, England

## Abstract

In the title compound, C_16_H_18_Cl_2_N^+^·Br^−^, the dihedral angle between the aromatic ring planes is 57.73 (5)°. In the absence of any strong hydrogen bonds, the structure results from a large number of competing weaker inter­actions including Cl⋯Cl [3.4610 (5) Å] and C—H⋯Cl contacts and both (aryl) C—H⋯Br and N^+^—C*sp* ^3^—H⋯Br^−^ cation–anion inter­actions.

## Related literature

Routes to quaternary ammonium compounds include the action of hexa­decyl halide on heterocycles such as pyridine (Shelton & Mariemont, 1942 ▶); the action of 1-haloalkanes and allied compounds on the higher alkyl esters of *p*-dimethyl­amino benzoic acid (Piggot & Woolvin, 1940 ▶); reaction of a terminal ep­oxy group with tertiary amine followed by the addition of an acid (Horst & Manfred, 1983 ▶); reaction of a tertiary amine, an alkyl­ating agent and an ep­oxy compound (Gary & Owen, 1991 ▶); reaction of an alkyl halide with pyridine or imidazole at 393 to 623 K (Kimihiko *et al.*, 2002 ▶); and reaction of tertiary amines, methanol and a cyclic ester (Walker, 2004 ▶). Quaternary ammonium compounds are utilized in many industrial processes, across a wide range of processes from sanitisers in detergent (Peng *et al*., 2002 ▶) to phase transfer catalysis (Stark *et al.*, 2004 ▶)). For Cl⋯Cl and C—H⋯Cl contacts, see: (López-Duplá, *et al.* 2003 ▶); (Desiraju & Steiner, 1999 ▶).
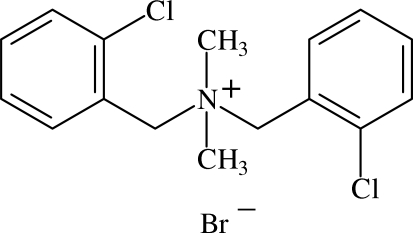


## Experimental

### 

#### Crystal data

C_16_H_18_Cl_2_N^+^·Br^−^
*M* *_r_* = 375.12
Monoclinic, 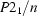

*a* = 11.9427 (5) Å
*b* = 8.9771 (4) Å
*c* = 15.0759 (6) Å
β = 97.411 (2)°
*V* = 1602.80 (12) Å^3^
*Z* = 4
Mo *K*α radiation
μ = 2.89 mm^−1^
*T* = 150 K
0.80 × 0.75 × 0.34 mm

#### Data collection

Bruker SMART 1000 CCD diffractometer
Absorption correction: multi-scan (*SADABS*; Sheldrick, 2003 ▶) *T*_min_ = 0.165, *T*_max_ = 0.376
13470 measured reflections
3816 independent reflections
3462 reflections with *I* > 2σ(*I*)
*R*_int_ = 0.015

#### Refinement

*R*[*F* ^2^ > 2σ(*F* ^2^)] = 0.019
*wR*(*F* ^2^) = 0.047
*S* = 1.03
3816 reflections
183 parameters
H-atom parameters constrained
Δρ_max_ = 0.39 e Å^−3^
Δρ_min_ = −0.21 e Å^−3^

### 

Data collection: *SMART* (Bruker, 2001 ▶); cell refinement: *SAINT* (Bruker, 2001 ▶); data reduction: *SAINT*; program(s) used to solve structure: *SHELXTL* (Sheldrick, 2008 ▶); program(s) used to refine structure: *SHELXTL*; molecular graphics: *SHELXTL*; software used to prepare material for publication: *SHELXTL* and local programs.

## Supplementary Material

Crystal structure: contains datablocks I, global. DOI: 10.1107/S160053680902159X/jh2078sup1.cif

Structure factors: contains datablocks I. DOI: 10.1107/S160053680902159X/jh2078Isup2.hkl

Additional supplementary materials:  crystallographic information; 3D view; checkCIF report

## Figures and Tables

**Table 1:** Hydrogen-bond geometry (Å, °)

*D*—H⋯*A*	*D*—H	H⋯*A*	*D*⋯*A*	*D*—H⋯*A*
C6—H6⋯Cl2^i^	0.95	2.87	3.6451 (15)	140
C9—H9*A*⋯Br1^ii^	0.98	2.99	3.6365 (13)	125
C9—H9*C*⋯Br1^iii^	0.98	2.95	3.8414 (15)	151
C7—H7*A*⋯Br1^iii^	0.99	2.66	3.6095 (13)	162
C7—H7*B*⋯Br1	0.99	2.99	3.8916 (13)	152
C10—H10*A*⋯Br1	0.99	2.75	3.6709 (13)	156
C16—H16⋯Br1^iv^	0.95	2.99	3.7361 (14)	136

Symmetry codes: (i) 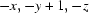; (ii) 
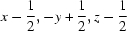
; (iii) 
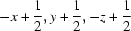
; (iv) 
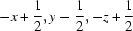
.

**Fig. 1.**
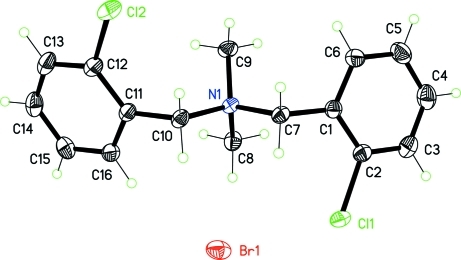
Perspective view of compound (I). Displacement elipsoids are drawn at the 50% level and H atoms are shown as spheres of arbitrary radius.

**Fig. 2.**
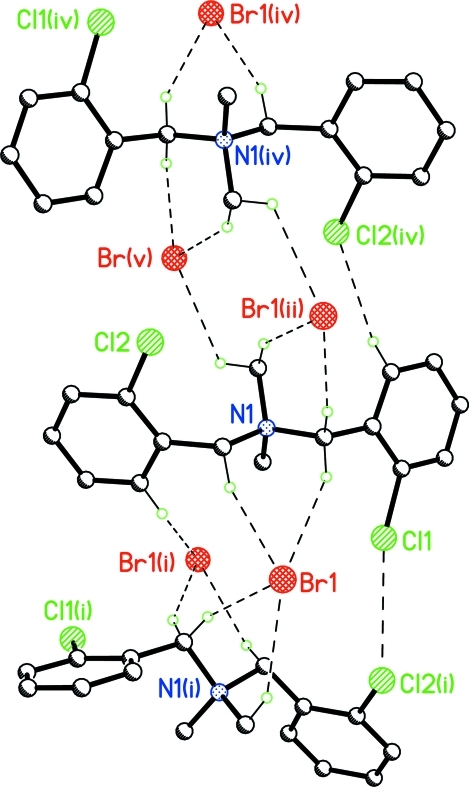
C — H···X— C, C — H···Br and Cl···Cl interactions. Symmetry codes: (i) -x + 1/2, y - 1/2, -z + 1/2; (ii) -x + 1/2, y + 1/2, -z + 1/2; (iv) -x, -y + 1, -z; (v) x - 1/2, -y + 1/2, z - 1/2.

**Fig. 3.**
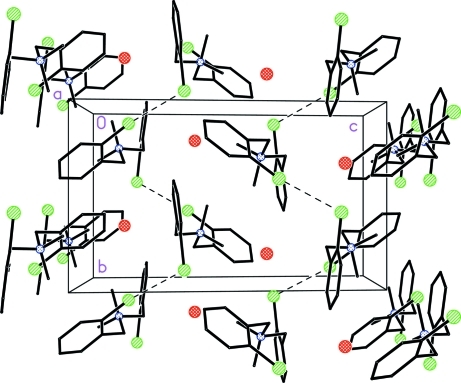
Packing diagram viewed down the a axis and showing the Cl···Cl interactions. Cl atoms are shown shaded, Br atoms are shown cross-hatched.

## supplementary crystallographic information

### Comment

Di(2-chlorobenzyl)dimethylammonium bromide (I, C_16_H_18_BrCl_2_N) was obtained simply and conveniently by reaction of 2-chlorobenzyl bromide with dimethylamine in the presence of triethylamine. It was characterized by IR and NMR spectroscopy and the stucture is shown in Fig. 1. The geometry at the central N atom is close to tetrahedral and the Cl atoms are *trans* to each other. The ions are linked through both Cl···Cl interactions and H-bonding involving both Cl and Br.

The intermolecular Cl1···Cl2 interaction at 3.4610 (5) Å (under symmetry operation (i), -*x* + 1/2, *y* - 1/2, -*z* + 1/2) is towards the lower end of the range (3.3029 (4) – 3.6759 (4) Å) observed in a related series of di- and trihaloanilinium halides (López-Duplá *et al.*, 2003). This interaction links the cations in zigzag chains running parallel to *b* (Fig. 2 and Fig. 3). Additionally there is a H-bond linking one of the aromatic carbon atoms (C6) to Cl2 (under symmetry operation (iv), -*x*, -*y* + 1, -*z*), while there are arguably six C—H···Br H-bonds linking each bromide anion to four cations (Table 2). Again these fall within the reported range (3.056 - 3.961 Å) (López-Duplá *et al.*, 2003), but only one involves an aromatic proton (on C16), the other five involve methyl or methylene groups adjacent to the N^+^ centre (N^+^— C(*sp*^3^)—H···Br^-^). This type of interaction is reasonably common (Desiraju & Steiner, 1999) and the geometry is as expected for H-bonding; however Desiraju and Steiner have pointed out that the primary interaction in such cases may be electrostatic attraction between the anion and the positive charge with the protons limiting the approach of the anion.

The four closest N^+^···Br^-^ distances are listed in table 1. The shortest pair are close to the mean value reported for contacts between a quaternary ammonium group and a bromide ion (Desiraju & Steiner, 1999); the remaining two distances are significantly longer and should probably be discounted, despite the fact that one of these molecules (symmetry operation (ii) -*x* + 1/2, *y* + 1/2, -*z* + 1/2) makes two "H-bonds" to Br1, suggesting a genuine N^+^— C(*sp*^3^)—H···Br^-^ attraction. In the absence of any strong H-bonds the structure is, of necessity, held together by a large number of competing weaker interactions between cations and between anions and cations.

### Experimental

2-Chlorobenzylbromide (0.65 ml, 1.0 mmol was dissolved in 25 ml dichloromethane and one ml of triethylamine was added, followed by dropwise addition of a solution of 33% dimethylamine (0.20 ml,1.5 mmol) in ethanol. The reaction mixture was stirred for eight hours, then neutralized with 10% sodium bicarbonate solution. The mixture was again stirred and the organic layer was separated, dried over anhydrous magnesium sulfate and filtered before being concentrated on a rotary evaporator. It was then cooled in a refrigerator to give the pure product as colorless needles in 43% yield. The presence of the quaternary ammonium species was established on the basis of the following spectroscopic data:

IR (KBr, cm^-1^): 3053 (s, ν_C—H_ aromatic), 2861 (m, ν_C—H_ aliphatic), 1600 (m, ν_C—C_), 776 (m, ν_C—Cl_), 1151 (m ν_C—N_) the latter band is characteristic of CH_2_—N(CH_3_)_2_. Absorption bands due to ν_C—Br_ and ν_N—H_ of the starting material in the region 690–515 cm^-1^(*m*) and 3400–3250 cm^-1^(*m*) respectively are absent.

NMR (CDCl_3_, p.p.m.. ^1^H): 3.26 (s, 6, CH_3_), 5.38 (s, 4, CH_2_), 7.48 – 7.56 (mult., 6, Ar), 8.16 (s, 2, Ar adjacent to Br).

### Refinement

H atoms were inserted at calculated positions and refined using a riding model. The constrained C—H distances were 0.95, 0.98 and 0.99 Å for aryl, methyl and methylene respectively. The H atoms of methylene and aryl groups were refined with *U*_iso_(H) = 1.2*U*_eq_(C) and those of the methyl groups with *U*_iso_(H) = 1.5*U*_eq_(C).

### Crystal data

**Table:**

C_16_H_18_Cl_2_N^+^·Br^−^	*F*(000) = 760
*M**_r_* = 375.12	*D*_x_ = 1.555 Mg m^−^^3^
Monoclinic, *P*2_1_/*n*	Mo *K*α radiation, λ = 0.71073 Å
Hall symbol: -P 2yn	Cell parameters from 8351 reflections
*a* = 11.9427 (5) Å	θ = 2.3–28.7°
*b* = 8.9771 (4) Å	µ = 2.89 mm^−^^1^
*c* = 15.0759 (6) Å	*T* = 150 K
β = 97.411 (2)°	Block, colourless
*V* = 1602.80 (12) Å^3^	0.80 × 0.75 × 0.34 mm
*Z* = 4	

### Data collection

**Table:**

Bruker SMART 1000 CCD diffractometer	3816 independent reflections
Radiation source: sealed tube	3462 reflections with *I* > 2σ(*I*)
graphite	*R*_int_ = 0.015
ω rotation with narrow frames scans	θ_max_ = 28.8°, θ_min_ = 2.1°
Absorption correction: multi-scan (*SADABS*; Sheldrick, 2003)	*h* = −15→15
*T*_min_ = 0.165, *T*_max_ = 0.376	*k* = −11→11
13470 measured reflections	*l* = −19→20

### Refinement

**Table:**

Refinement on *F*^2^	Primary atom site location: structure-invariant direct methods
Least-squares matrix: full	Secondary atom site location: difference Fourier map
*R*[*F*^2^ > 2σ(*F*^2^)] = 0.019	Hydrogen site location: inferred from neighbouring sites
*wR*(*F*^2^) = 0.047	H-atom parameters constrained
*S* = 1.02	*w* = 1/[σ^2^(*F*_o_^2^) + (0.0215*P*)^2^ + 0.7575*P*] where *P* = (*F*_o_^2^ + 2*F*_c_^2^)/3
3816 reflections	(Δ/σ)_max_ = 0.001
183 parameters	Δρ_max_ = 0.39 e Å^−^^3^
0 restraints	Δρ_min_ = −0.21 e Å^−^^3^

### Special details

**Table:**

Geometry. All e.s.d.'s (except the e.s.d. in the dihedral angle between two l.s. planes) are estimated using the full covariance matrix. The cell e.s.d.'s are taken into account individually in the estimation of e.s.d.'s in distances, angles and torsion angles; correlations between e.s.d.'s in cell parameters are only used when they are defined by crystal symmetry. An approximate (isotropic) treatment of cell e.s.d.'s is used for estimating e.s.d.'s involving l.s. planes.
Refinement. Refinement of *F*^2^ against ALL reflections. The weighted *R*-factor *wR* and goodness of fit *S* are based on *F*^2^, conventional *R*-factors *R* are based on *F*, with *F* set to zero for negative *F*^2^. The threshold expression of *F*^2^ > σ(*F*^2^) is used only for calculating *R*-factors(gt) *etc*. and is not relevant to the choice of reflections for refinement. *R*-factors based on *F*^2^ are statistically about twice as large as those based on *F*, and *R*- factors based on ALL data will be even larger.

### Fractional atomic coordinates and isotropic or equivalent isotropic displacement parameters (Å^2^)

**Table:**

	*x*	*y*	*z*	*U*_iso_*/*U*_eq_	
C1	0.35722 (11)	0.30048 (15)	0.05726 (9)	0.0197 (3)	
C2	0.45188 (11)	0.20813 (15)	0.06312 (9)	0.0215 (3)	
Cl1	0.48415 (3)	0.09081 (4)	0.15526 (2)	0.02628 (8)	
C3	0.52466 (12)	0.20869 (18)	−0.00141 (10)	0.0280 (3)	
H3	0.5886	0.1449	0.0042	0.034*	
C4	0.50319 (14)	0.30335 (19)	−0.07430 (10)	0.0329 (3)	
H4	0.5517	0.3031	−0.1195	0.039*	
C5	0.41118 (14)	0.39814 (19)	−0.08124 (10)	0.0326 (3)	
H5	0.3967	0.4632	−0.1311	0.039*	
C6	0.33996 (12)	0.39827 (17)	−0.01548 (10)	0.0259 (3)	
H6	0.2784	0.4660	−0.0199	0.031*	
C7	0.27893 (11)	0.30423 (14)	0.12803 (9)	0.0184 (3)	
H7A	0.2604	0.4093	0.1397	0.022*	
H7B	0.3191	0.2626	0.1841	0.022*	
N1	0.16846 (9)	0.21772 (12)	0.10372 (7)	0.0172 (2)	
C8	0.19294 (12)	0.05980 (15)	0.08053 (10)	0.0231 (3)	
H8A	0.2367	0.0585	0.0298	0.035*	
H8B	0.1217	0.0062	0.0644	0.035*	
H8C	0.2364	0.0114	0.1322	0.035*	
C9	0.09604 (12)	0.28868 (16)	0.02635 (9)	0.0226 (3)	
H9A	0.0224	0.2389	0.0171	0.034*	
H9B	0.1330	0.2791	−0.0278	0.034*	
H9C	0.0854	0.3944	0.0392	0.034*	
C10	0.10924 (11)	0.22455 (16)	0.18762 (9)	0.0201 (3)	
H10A	0.1597	0.1804	0.2380	0.024*	
H10B	0.0975	0.3304	0.2024	0.024*	
C11	−0.00317 (11)	0.14542 (16)	0.17982 (9)	0.0201 (3)	
C12	−0.10616 (12)	0.22153 (16)	0.16489 (9)	0.0221 (3)	
Cl2	−0.10838 (3)	0.41433 (4)	0.15277 (2)	0.02902 (8)	
C13	−0.20899 (12)	0.14761 (19)	0.16070 (10)	0.0287 (3)	
H13	−0.2777	0.2019	0.1503	0.034*	
C14	−0.21075 (13)	−0.00522 (19)	0.17171 (10)	0.0306 (3)	
H14	−0.2808	−0.0565	0.1679	0.037*	
C15	−0.11021 (13)	−0.08362 (18)	0.18827 (10)	0.0288 (3)	
H15	−0.1113	−0.1885	0.1964	0.035*	
C16	−0.00792 (12)	−0.00860 (17)	0.19297 (9)	0.0244 (3)	
H16	0.0605	−0.0632	0.2054	0.029*	
Br1	0.332790 (12)	0.178154 (15)	0.376859 (9)	0.02633 (5)	

### Atomic displacement parameters (Å^2^)

**Table:**

	*U*^11^	*U*^22^	*U*^33^	*U*^12^	*U*^13^	*U*^23^
C1	0.0179 (6)	0.0197 (6)	0.0211 (6)	−0.0019 (5)	0.0009 (5)	−0.0006 (5)
C2	0.0199 (6)	0.0226 (7)	0.0212 (6)	0.0005 (5)	0.0000 (5)	−0.0001 (5)
Cl1	0.02153 (16)	0.02815 (18)	0.02852 (17)	0.00756 (13)	0.00083 (13)	0.00558 (14)
C3	0.0215 (7)	0.0344 (8)	0.0284 (7)	0.0010 (6)	0.0042 (6)	−0.0048 (6)
C4	0.0314 (8)	0.0444 (9)	0.0244 (7)	−0.0066 (7)	0.0096 (6)	−0.0029 (7)
C5	0.0354 (8)	0.0375 (9)	0.0244 (7)	−0.0061 (7)	0.0024 (6)	0.0081 (6)
C6	0.0234 (7)	0.0258 (7)	0.0275 (7)	−0.0007 (6)	−0.0003 (6)	0.0057 (6)
C7	0.0163 (6)	0.0182 (6)	0.0200 (6)	0.0006 (5)	−0.0003 (5)	−0.0012 (5)
N1	0.0153 (5)	0.0179 (5)	0.0179 (5)	0.0021 (4)	0.0006 (4)	−0.0008 (4)
C8	0.0222 (7)	0.0174 (6)	0.0306 (7)	0.0005 (5)	0.0072 (5)	−0.0040 (5)
C9	0.0206 (7)	0.0279 (7)	0.0178 (6)	0.0025 (5)	−0.0032 (5)	0.0017 (5)
C10	0.0163 (6)	0.0274 (7)	0.0165 (6)	0.0027 (5)	0.0012 (5)	−0.0019 (5)
C11	0.0174 (6)	0.0280 (7)	0.0150 (6)	0.0026 (5)	0.0019 (5)	−0.0012 (5)
C12	0.0200 (7)	0.0278 (7)	0.0180 (6)	0.0038 (5)	0.0011 (5)	−0.0033 (5)
Cl2	0.02630 (17)	0.02741 (18)	0.03270 (19)	0.00973 (14)	0.00138 (14)	−0.00288 (14)
C13	0.0171 (7)	0.0423 (9)	0.0265 (7)	0.0025 (6)	0.0022 (5)	−0.0050 (6)
C14	0.0232 (7)	0.0420 (9)	0.0272 (7)	−0.0083 (6)	0.0058 (6)	−0.0055 (6)
C15	0.0348 (8)	0.0288 (8)	0.0244 (7)	−0.0035 (6)	0.0098 (6)	−0.0008 (6)
C16	0.0238 (7)	0.0289 (7)	0.0210 (7)	0.0053 (6)	0.0056 (5)	0.0026 (6)
Br1	0.02822 (8)	0.02006 (7)	0.02794 (8)	−0.00575 (5)	−0.00691 (5)	0.00494 (5)

### Geometric parameters (Å, °)

**Table:**

C1—C2	1.3955 (19)	C9—H9A	0.9800
C1—C6	1.3990 (19)	C9—H9B	0.9800
C1—C7	1.5068 (18)	C9—H9C	0.9800
C2—C3	1.386 (2)	C10—C11	1.5100 (18)
C2—Cl1	1.7463 (14)	C10—H10A	0.9900
C3—C4	1.387 (2)	C10—H10B	0.9900
C3—H3	0.9500	C11—C16	1.399 (2)
C4—C5	1.383 (2)	C11—C12	1.3995 (18)
C4—H4	0.9500	C12—C13	1.390 (2)
C5—C6	1.387 (2)	C12—Cl2	1.7403 (15)
C5—H5	0.9500	C13—C14	1.382 (2)
C6—H6	0.9500	C13—H13	0.9500
C7—N1	1.5341 (16)	C14—C15	1.386 (2)
C7—H7A	0.9900	C14—H14	0.9500
C7—H7B	0.9900	C15—C16	1.389 (2)
N1—C8	1.4979 (16)	C15—H15	0.9500
N1—C9	1.5014 (16)	C16—H16	0.9500
N1—C10	1.5279 (16)	Br1—N1	4.3416 (11)
C8—H8A	0.9800	Br1—N1^i^	4.1439 (11)
C8—H8B	0.9800	Br1—N1^ii^	4.8527 (11)
C8—H8C	0.9800	Br1—N1^iii^	5.0105 (11)
			
C2—C1—C6	117.29 (13)	N1—C8—H8C	109.5
C2—C1—C7	122.68 (12)	H8A—C8—H8C	109.5
C6—C1—C7	119.92 (12)	H8B—C8—H8C	109.5
C3—C2—C1	122.00 (13)	N1—C9—H9A	109.5
C3—C2—Cl1	117.81 (11)	N1—C9—H9B	109.5
C1—C2—Cl1	120.17 (10)	H9A—C9—H9B	109.5
C2—C3—C4	119.33 (14)	N1—C9—H9C	109.5
C2—C3—H3	120.3	H9A—C9—H9C	109.5
C4—C3—H3	120.3	H9B—C9—H9C	109.5
C5—C4—C3	120.07 (14)	C11—C10—N1	114.74 (10)
C5—C4—H4	120.0	C11—C10—H10A	108.6
C3—C4—H4	120.0	N1—C10—H10A	108.6
C4—C5—C6	120.05 (14)	C11—C10—H10B	108.6
C4—C5—H5	120.0	N1—C10—H10B	108.6
C6—C5—H5	120.0	H10A—C10—H10B	107.6
C5—C6—C1	121.19 (14)	C16—C11—C12	117.03 (13)
C5—C6—H6	119.4	C16—C11—C10	120.39 (12)
C1—C6—H6	119.4	C12—C11—C10	122.47 (13)
C1—C7—N1	114.37 (10)	C13—C12—C11	121.81 (14)
C1—C7—H7A	108.7	C13—C12—Cl2	117.94 (11)
N1—C7—H7A	108.7	C11—C12—Cl2	120.24 (11)
C1—C7—H7B	108.7	C14—C13—C12	119.69 (14)
N1—C7—H7B	108.7	C14—C13—H13	120.2
H7A—C7—H7B	107.6	C12—C13—H13	120.2
C8—N1—C9	109.29 (10)	C13—C14—C15	119.95 (14)
C8—N1—C10	110.88 (10)	C13—C14—H14	120.0
C9—N1—C10	110.14 (10)	C15—C14—H14	120.0
C8—N1—C7	110.31 (10)	C14—C15—C16	119.92 (14)
C9—N1—C7	111.24 (10)	C14—C15—H15	120.0
C10—N1—C7	104.93 (9)	C16—C15—H15	120.0
N1—C8—H8A	109.5	C15—C16—C11	121.57 (13)
N1—C8—H8B	109.5	C15—C16—H16	119.2
H8A—C8—H8B	109.5	C11—C16—H16	119.2
			
C6—C1—C2—C3	−2.0 (2)	C8—N1—C10—C11	−60.79 (14)
C7—C1—C2—C3	−178.34 (13)	C9—N1—C10—C11	60.32 (15)
C6—C1—C2—Cl1	176.20 (11)	C7—N1—C10—C11	−179.87 (11)
C7—C1—C2—Cl1	−0.19 (18)	N1—C10—C11—C16	82.49 (15)
C1—C2—C3—C4	−0.1 (2)	N1—C10—C11—C12	−101.51 (15)
Cl1—C2—C3—C4	−178.34 (12)	C16—C11—C12—C13	−1.8 (2)
C2—C3—C4—C5	1.3 (2)	C10—C11—C12—C13	−177.91 (13)
C3—C4—C5—C6	−0.3 (2)	C16—C11—C12—Cl2	177.16 (10)
C4—C5—C6—C1	−2.0 (2)	C10—C11—C12—Cl2	1.03 (18)
C2—C1—C6—C5	3.0 (2)	C11—C12—C13—C14	0.2 (2)
C7—C1—C6—C5	179.49 (13)	Cl2—C12—C13—C14	−178.79 (12)
C2—C1—C7—N1	−101.76 (14)	C12—C13—C14—C15	1.0 (2)
C6—C1—C7—N1	81.94 (15)	C13—C14—C15—C16	−0.6 (2)
C1—C7—N1—C8	55.23 (14)	C14—C15—C16—C11	−1.1 (2)
C1—C7—N1—C9	−66.23 (14)	C12—C11—C16—C15	2.2 (2)
C1—C7—N1—C10	174.70 (11)	C10—C11—C16—C15	178.46 (12)

Symmetry codes: (i) −*x*+1/2, *y*−1/2, −*z*+1/2; (ii) −*x*+1/2, *y*+1/2, −*z*+1/2; (iii) *x*+1/2, −*y*+1/2, *z*+1/2.

### Hydrogen-bond geometry (Å, °)

**Table:**

*D*—H···*A*	*D*—H	H···*A*	*D*···*A*	*D*—H···*A*
C6—H6···Cl2^iv^	0.95	2.87	3.6451 (15)	140
C9—H9A···Br1^v^	0.98	2.99	3.6365 (13)	125
C9—H9C···Br1^ii^	0.98	2.95	3.8414 (15)	151
C7—H7A···Br1^ii^	0.99	2.66	3.6095 (13)	162
C7—H7B···Br1	0.99	2.99	3.8916 (13)	152
C10—H10A···Br1	0.99	2.75	3.6709 (13)	156
C16—H16···Br1^i^	0.95	2.99	3.7361 (14)	136

Symmetry codes: (iv) −*x*, −*y*+1, −*z*; (v) *x*−1/2, −*y*+1/2, *z*−1/2; (ii) −*x*+1/2, *y*+1/2, −*z*+1/2; (i) −*x*+1/2, *y*−1/2, −*z*+1/2.
